# Postoperative raised ICP after minimally invasive surgery for monosutural craniosynostosis

**DOI:** 10.1007/s00381-026-07289-7

**Published:** 2026-05-09

**Authors:** Renata Martinelli, Miriana Cusumano, Gianpiero Tamburrini, Luca Massimi

**Affiliations:** 1 Pediatric Neurosurgery, Fondazione Policlinico Universitario A. Gemelli IRCCS, Rome, Italy; 2 Department of Neuroscience, Università Cattolica del Sacro Cuore, Rome, Italy

**Keywords:** Craniosynostosis, Intracranial pressure, Minimally invasive surgery, Strip craniectomies, Scaphocephaly

## Abstract

**Introduction:**

Minimally invasive craniectomies (MIC) for monosutural craniosynostosis are more and more used. Their results are usually investigated in terms of surgical, esthetic, and neuropsychological outcomes but not specifically about the risk of postoperative raised intracranial pressure (ICP). The goal of this article is to review the literature and the personal experience looking for the possible risk of postoperative raised ICP after MIC.

**Methods:**

A systematic review adhering to PRISMA guidelines was realized. In parallel, the personal experience concerning scaphocephalic children consecutively operated on between 2010 and 2013 by MIC was analyzed. Children operated on by open cranial vault remodeling (OCVR) in the same period were considered as controls.

**Results:**

Overall, 3048 patients from 14 studies were included, 5% of them requiring a reoperation after a 59.9-month mean follow-up. A total of 2297 children were treated by MIC: 2.8% (65 cases) showed postoperative raised ICP, the rate of reoperation was 4.4% (102 cases). The risk of reoperation varies across the techniques, being higher in modified or spring-assisted strip craniectomies (2.4–14.6%) than in endoscopy-assisted craniectomies (2.4%). Among 751 cases treated by OCVR, this risk varied from 0.95 to 7.1%. Also in the personal experience, no significant differences were found between MIC (1.5% of reoperation for raised ICP) and OCVR (0.8%).

**Conclusions:**

The risk of postoperative raised ICP seems to be relatively low after surgery for monosutural synostosis without a significant difference between MIC and OCVR. Missing standardized methods for postoperative screening, missing information about the reason of reoperation in some studies, and the retrospective analysis methods in most of them are the main limitations of this study.

## Introduction

Introduced about 30 years ago, mini-invasive procedures are still gaining a growing consensus for the correction of monosutural craniosynostosis. Moreover, the trend is towards less and less invasive procedures, as demonstrated by a study provided by Da Costa and coworkers [12]. In this review and meta-analysis on 1564 children operated on with different endoscopic mini-invasive techniques, indeed, the less invasive operations (suturectomies < 2 cm, no lateral osteotomies) were comparable with more invasive procedures (suturectomies > 2 cm plus lateral osteotomies) in terms of cephalic index (CI) gain and helmet therapy duration but with a lower risk of blood transfusion.

Currently, the postoperative results in patients treated for craniosynostosis are usually assessed in terms of (1) surgical outcome: namely cephalic index [6, 15, 54] or other cephalometric parameters changes [27, 37, 49], transfusion rate/blood loss [10, 34, 50], length of hospital stay [24, 45] or helmet therapy need/duration [13, 26]; 2) cosmetic outcome [3, 23, 55]; 3) neurodevelopmental or other functional outcomes [8, 20, 36, 39]. On the other hand, however, a systematic assessment of the outcome in term of risk of postoperative intracranial hypertension (the prevention of which is one of the goals of surgery especially in sagittal craniosynostosis) is missing. The aim of the present review is to provide information about the occurrence of raised intracranial pressure (ICP) after mini-invasive surgery for monosutural synostoses.

## Methods

### Review

This systematic review adhered to PRISMA (Preferred Reporting Items for Systematic Reviews and Meta-Analyses) guidelines, for identifying and critically evaluating relevant studies. All procedures followed the protocols outlined in the Cochrane Handbook of Systematic Reviews and Meta-analysis of Interventions (version 6.3) [11, 35]. The review question was structured using the PICO (Population, Intervention, Comparison, Outcomes) framework: “In patients with craniosynostosis (P) undergoing minimal invasive surgery (I) and subjected to postoperative clinical and instrumental evaluations (C), is the risk of postoperative intracranial hypertension higher than in other procedures (O)?” [46]. One electronic database (PubMed) was evaluated using comprehensive search terms: ((“Craniosynostoses” OR craniosynostosis OR craniosynostoses) AND (“Minimally Invasive Surgical Procedures” OR “minimally invasive” OR “endoscopic” OR “endoscopy” OR “endoscopic-assisted” OR “endoscope-assisted” OR “endoscopic surgery” OR “strip craniectomy” OR “endoscopic strip craniectomy”)) AND (“Intracranial Pressure” OR “intracranial hypertension” OR “raised ICP” OR “elevated intracranial pressure” OR complication OR risk).” The latest research was conducted in August 2025. Two authors (R.M. and L.M.) independently conducted the abstract screening for eligibility. Studies meeting the following criteria were included: studies referring to at least one pediatric patient with craniostenosis who underwent minimally invasive craniectomy (MIC), in which ICP was assessed in the postoperative period. Exclusion criteria comprised studies published in languages other than English, reviews and meta-analyses, case reports, letters to the editor, and studies assessing efficacy of minimally invasive techniques but not mentioning ICP evaluation. Extracted data encompassed study characteristics, patient demographics, type of craniosynostosis, surgical technique, and outcomes. In studies that did not use a quantitative method to measure ICP, clinical signs, fundoscopy, and imaging were considered equivalent indicators of raised ICP. 

### Personal experience

Herein, we addressed only sagittal craniosynostoses either because of the higher risk of developing raised ICP or because, in our institution, we routinely use MIC only in children with scaphocephaly. The technique, consisting of suturectomies and partial remodeling without postoperative helmeting in children less than 5–6 months, has been described elsewhere [29]. For the present analysis, we considered the 2010–2023 time period to have homogeneous and complete data on children consecutively operated on and to get a 2-year minimum follow-up. The statistical analysis was carried out through the chi-square statistic and the Student *t*-test. *P* < 0.05 was considered a statistically significant difference. Ethical approval was waived because of the retrospective nature of the study and all procedures being part of the routine surgical care.

## Results

### Review

**Fig. 1:**
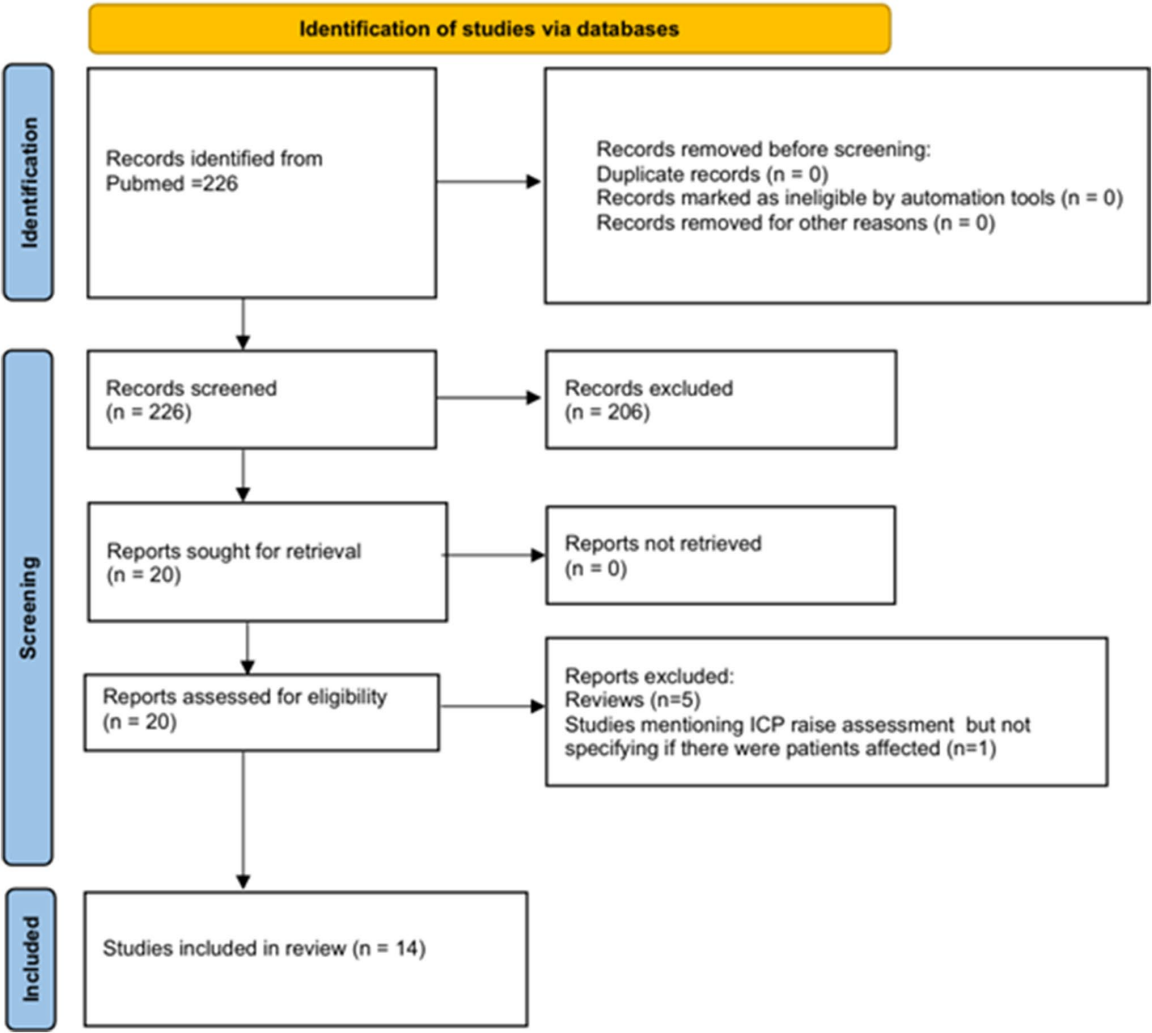
PRISMA flow chart

The search of the literature on PubMed yielded a total of 226 results which were carefully screened, and a total of 205 records were excluded. Ultimately 14 studies were found to be relevant for the present research and were assessed (Fig. 1), including 12 retrospective studies and 2 prospective studies (Table 1).

**Table 1:** Studies included in this review

Author, year	Type of study
Labuschagne et al., 2023 [25]	Retrospective
Yousefi et al., 2023 [56]	Retrospective
Riordan et al., 2020 [41]	Retrospective
Abdel-Alim et al., 2020 [1]	Retrospective
Dalle Ore et al., 2018 [14]	Retrospective
Thomas et al., 2015 [51]	Retrospective
Van Veelen et al., 2018 [53]	Prospective
Makoshi et al., 2025 [28]	Prospective
Esparza et al., 2008 [17]	Retrospective
Arts et al., 2018 [5]	Retrospective
Mathijssen et al., 2021 [31]	Retrospective
Van Veelen et al., 2013 [52]	Retrospective
Rodgers et al., 2017 [42]	Retrospective
Sadon et al., 2025 [44]	Retrospective

A total of 3048 patients were included, with 2297 children undergoing minimally invasive procedures, and 751 undergoing open cranial vault remodeling (OCVR) or other invasive procedures. The mean age at surgery was 4.4 months, with only one study not reporting demographics. Half of the studies (*n* = 7) focused on all types of craniosynostosis, while the other 7 studies focused on outcomes only for sagittal craniosynostosis (Table 2). The total mean follow-up was 59.9 months, with one study not specifying the mean follow-up period [17].

**Table 2:** Patients demographics

Author, year	N. of patients (MIC)	Mean age at surgery (months)	Type of craniosynostosis (MIC and open)
Labuschagne et al., 2023 [25]	18	3.4	Sagittal (18)
Yousefi et al., 2023 [56]	10	1.3	Multisuture (6), syndromic (4)
Riordan et al., 2020 [41]	500	3.0	Sagittal (252), metopic (95), unicoronal (79), bicoronal (42), lambdoid (12), multisuture (20) syndromic (34)
Abdel-Alim et al., 2020 [1]	218	< 12	Sagittal (218)
Dalle Ore et al., 2018 [14]	235	3.8	Sagittal (190), metopic (32), unicoronal (11), lambdoid (2)
Thomas et al., 2015 [51]	89	6	Sagittal (217)
Van Veelen et al., 2018 [53]	83	5.8	Sagittal (83)
Makoshi et al., 2025 [28]	52	5.3	Sagittal (18), metopic (15), unicoronal (7), lambdoid (6), multisuture (6)
Esparza et al., 2008 [17]	88	6.7	Sagittal (155), metopic (50), unicoronal (28), lambdoid (1) multisuture (20), syndromic (31)
Arts et al., 2018 [5]	121	3.9	Sagittal (93), metopic (50), unicoronal/lambdoid (26), bicoronal (3), multisuture (6) syndromic (9)
Mathijssen et al., 2021 [21]	418	NA	Sagittal (378), metopic (192), unicoronal (84), bicoronal (48) lambdoid (14), multisuture (70)
Van Veelen et al., 2013 [52]	79	3.8	Sagittal (79)
Rodgers et al., 2017 [42]	100	5.6	Sagittal (100)
Sadon et al., 2025 [44]	286	3.1	Sagittal (286)

A total of 151 out of 3048 patients underwent a second surgical procedure (5%). Specifically, among the 2297 patients treated with MICs, 102 required a reoperation, yielding a reoperation rate of 4.4%. Raised ICP was reported in 69 out of 3048 patients (2.2%), including 65 out of 2297 cases within the MIC cohort (2.8%). One study did not clearly specify the assignment of raised ICP cases and patients requiring second surgery to a specific surgical cohort, and this data was therefore excluded from analysis [17].

The methods used to assess ICP were heterogeneous across studies, including clinical evaluation, suggestive neuroimaging, fundoscopic examination with evidence of papilledema, and, in 7 studies, direct invasive ICP monitoring, with variable monitoring durations and threshold definitions (Table 3). Specifically, in 6 studies, fundoscopy, clinical signs, and/or imaging were evaluated without invasive ICP monitoring and were therefore initially considered equivalent indicators of intracranial hypertension. Among these, only 2 studies that assessed fundoscopy without confirmation via invasive ICP monitoring eventually reported the presence of patients with postoperative intracranial hypertension (Table 3).

**Table 3:** Pooled data regarding type of surgery, reoperation rate, ICP raise and monitoring, and mean follow up

Author, year	N. of patients	Surgical technique	Reoperation rate	N. of patients with postoperative intracranial hypertension	Method of ICP measurement	ICP values	Mean FUP (months)
Labuschagne et al., 2023 [25]	18	EASC + helmet	0%	0	Clinical, fundoscopy, imaging	NA	17 (range 12–28)
Yousefi et al., 2023 [56]	10	EASC + helmet	1 (10%)	0	Clinical, imaging	NA	38.9 (range 24–48)
Riordan et al., 2020 [41]	500	EASC + helmet	ESC: 2 (6.1%); OCVR: 1 (3.4%) 15 (3%)	12 (2.4%)	Clinical, imaging	NA	67.2
Abdel-Alim et al., 2020 [1]	218	FBR, ESC, SAC	15 (5 FBR, 8 ESC, 2 SAC)	6 (1 FBR, 4 ESC, 1 SAC) (2.7%)	Fundoscopy, invasive intraparenchymal ICP sensor	NA	72
Dalle Ore et al., 2018 [14]	235	EASC + helmet	7 (3%)	0	Clinical, fundoscopy	NA	33.6
Thomas et al., 2015 [51]	MSC: 89; OCVR: 128	MSC; OCVR	MSC: 19 (21.3%); CR: 2 (1.6%)	MSC: 13 (14.6%); CR: 2 (1.6%)	clinical, fundoscopy, imaging, invasive intraparenchymal ICP sensor	≥ 15 mmHg	86 (range 24–212)
Van Veelen et al., 2018 [53]	83	MSC + SAC	1 (1.2%)	2 (2.4%)	Fundoscopy	NA	39.8
Makoshi et al., 2025 [28]	52	EASC	0%	0	Invasive intraparenchymal ICP sensor	Mean ICP 12.7–2.9 mmHg	2.58 ± 4.78 (range 4 days–24)
Esparza et al., 2008 [17]	EASC/MSC: 88; OCVR: 195	EASC; MSC; OCVR	37 (11.5%)	25 (NA)	Invasive intraparenchymal ICP sensor, imaging, clinical	NA	NA
Arts et al., 2018 [5]	EASC: 121; OCVR: 66	EASC; OCVR	EACS: 4 (3.3%); OCVR: 4 (6.1%)	EACS: 2 (1.6%); OCVR: 2 (3%)	Invasive intraparenchymal ICP sensor (1), clinical	22–24 mmHg lying, 8 mmHg sitting	12
Mathijssen et al., 2021 [21]	MICs: 418; others: 362	MSC + helmet, FBR or SAC	MICs: 29 (6.9%); others: 6 (1.6%)	MICs: 18 (4.3%)	Fundoscopy, imaging, clinical	NA	216
Van Veelen et al., 2013 [52]	79	ESC + barrel staves	4 (5%)	7 (9%)	Fundoscopy, imaging, invasive intraparenchymal ICP sensor	≥ 15 mmHg	46.8
Rodgers et al., 2017 [42]	100	SAC	5 (5%)	2 (2%)	NA	NA	38 (range 11–65)
Sadon et al., 2025 [44]	286	MSC	2 (0.7%)	3 (1%)	Fundoscopy + invasive intraparenchymal ICP sensor	(1) 5–26 mmHg (borderline) (2) 28–41 mmHg (3) 24–30 mmHg	109.2

*EASC*, endoscopic-assisted strip craniectomy; *OCVR*, open cranial vault remodeling; *FBR*, frontobiparietal remodeling; *ESC*, extended strip craniectomy; *SAC*, spring-assisted correction; *FOA*, fronto-orbital advancement; *MICs*, minimally invasive craniectomies; *MSC*, modified or mini-invasive strip craniectomy

Analyzing the data available regarding craniosynostosis subtypes, sagittal craniosynostosis was by far the most frequently represented type, accounting for 2087 patients, 4.3% of whom needed a second surgery. The highest relative reoperation rate was in the bi-coronal craniosynostosis cohort (11.8%). The available data did not allow us to determine the exact number of patients, within each specific craniosynostosis subtype, who developed postoperative raised intracranial pressure (Table 4).

**Table 4:** Incidence of reoperations according to craniosynostosis type

Craniosynostosis type	N. of patients	Patients reoperated
Sagittal	2087	90 (4.3%)
Metopic	434	18 (4.1%)
Unicoronal	222	16 (7.2%)
Bicoronal	93	11 (11.8%)
Lambdoid	48	3 (6.3%)
Multisuture	128	12 (9.4%)
Syndromic	78	7 (9%)

### Personal experience

**Fig. 2:**
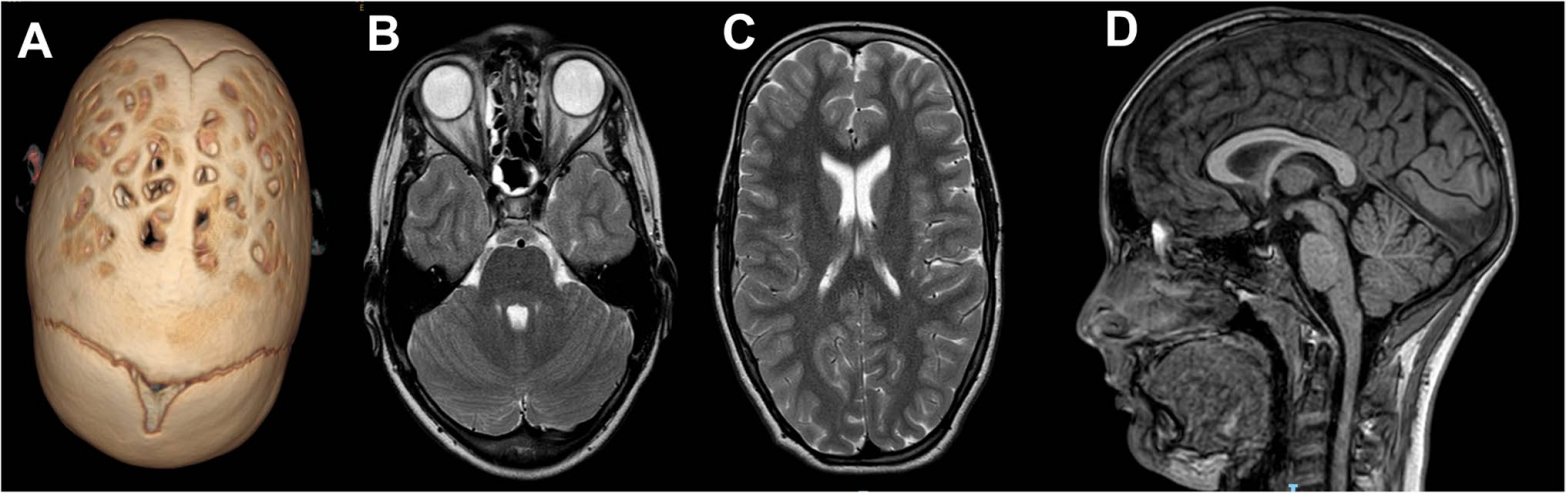
Preoperative 3D CT scan (**A**) of a young boy undergoing mini-invasive surgery at the age of 4 months. Postoperative MRI (**B**–**D**), performed 4.5 years later because of papilledema, does not show relevant findings

**Fig. 3:**
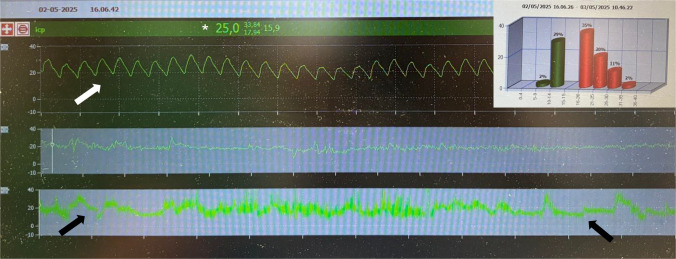
ICP recording of one young boy of Fig. 2. The ICP curve shape (white arrow) as well as the ICP trend in the 24 h (black arrows) and the ICP values (asterisk) is altered, indicating raised ICP. In the box, a summary of the recording during 48 h is reported (normal values set at ≤ 15 mmHg): ICP is > 16 mmHg for 68% of the recording time

Overall, 255 scaphocephalic children (group A) were operated on by MIC in the aforementioned period. The M/F ratio was 3.4 and the mean age at surgery was 4.24 months (range: 2–9 months). After a 7-year mean follow-up, 6 children (2.3%) needed a reoperation: 2 of them because of cosmetic problems (0.7%) and the remaining 4 because of raised ICP (1.5%). During the follow-up period, 7 children were found to present papilledema, according to routine postoperative investigation, but only one showed symptoms (cough headache). At this time, all of them received a CT scan, which excluded a late multi-sutural synostosis in all cases, and an MRI, which ruled out other possible causes (e.g., hydrocephalus, tumors) or consequences of raised ICP (e.g., secondary cerebellar tonsils descent) in all cases (Fig. 2). A genetic syndrome was ruled out in all cases. Moreover, according to our protocol [48], all those 7 patients underwent prolonged ICP recording. Only 4 out of 7 children showed abnormal recording (Fig. 3) and, therefore, were reoperated on by OCVR. The remaining 3 children are still followed up (papilledema regressed in 2 of them).

**Fig. 4:**
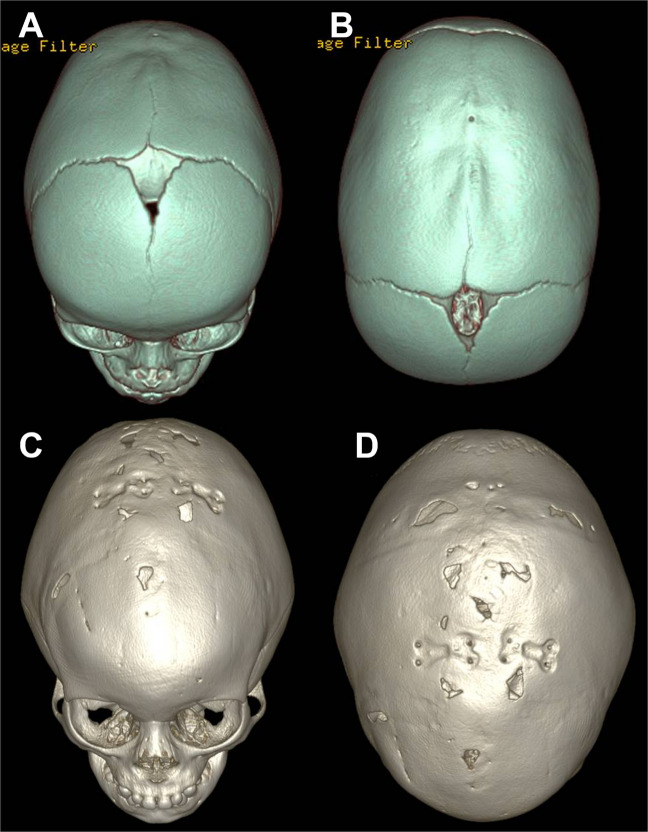
**A**, **B** Preoperative 3D CT scan, performed at the age of 5 months, of a young girl operated on by cranial vault remodeling when she was 8.5 months old. The exam shows sagittal craniosynostosis. **C**, **D** Postoperative 3D CT scan, performed 11 months after surgery because of papilledema, demonstrating restenosis with involvement of the metopic (with “trigonocephalic” frontal bossing) and bilateral coronal sutures

In the same period, 2 out of 112 scaphocephalic children treated with OCVR (Group B) were reoperated on because of late raised ICP (1.7%). In this instance, one of them showed progression of the synostosis on CT scan (Fig. 4). Therefore, in this group (M/F ratio: 3.7; mean age at surgery: 6.8 months (range: 4–60 months)), the reoperation rate for “pure” sagittal synostosis accounted for 0.8% (1/112 patients).

A statistically significant difference between the 2 groups was found for the age at surgery, which, as expected, was relevantly lower in group A (*P* = 0.0001). No statistically significant differences were found for the number of patients reoperated on (6 vs 2 cases), number of patients reoperated on because of raised ICP (4 vs 2 cases), number of patients reoperated on because of raised ICP and pure scaphocephaly (4 vs 1 cases), and number of patients with papilledema in the postoperative period (7 vs 2 cases).

## Discussion

This systematic review analyzed 14 studies encompassing 2297 patients who underwent MICs for craniosynostosis, of whom 65 developed postoperative intracranial hypertension (2.8%). These results are clinically relevant, especially considering that elevated ICP in children often lacks classic symptoms such as headache, altered consciousness, nausea, or vomiting and may therefore go undetected, leading to long-term sequelae such as cognitive deterioration and visual loss [9, 16]. Although the literature on the long-term neuropsychological consequences of elevated ICP remains inconclusive, an inverse relationship between ICP levels and cognitive function in patients with unilateral coronal synostosis was demonstrated, with an improvement after craniosynostosis correction, especially if performed before 6 months of age [4, 38, 40].

The risk of postoperative intracranial hypertension can be evaluated with invasive ICP monitoring, which is considered the gold standard for diagnosis but presents several limitations: its invasive nature makes it unsuitable as a screening tool, and there is still no standardized threshold for pathological ICP values in pediatric patients. Although a mean ICP > 15 mmHg is generally considered pathological, many children present with borderline values (10–15 mmHg) particularly during sleep, and in such cases, abnormal waveform patterns may also indicate pathologic ICP [9, 48].

The interpretation of papilledema represents an additional critical issue. Although papilledema is commonly used as a surrogate marker of elevated ICP, it does not consistently correlate with intracranial hypertension, particularly in pediatric populations and younger patients: papilledema may be absent in early or moderate ICP raise and can show poor correlation with actual ICP values. Furthermore, its development may be delayed and may persist even after normalization of ICP, leading to potential misclassification. The examination is also highly operator-dependent and technically challenging in young children, resulting in variability in detection [24, 28]. Furthermore, two studies included in this review relied on fundoscopic findings without confirmatory invasive monitoring and still reported cases of postoperative intracranial hypertension. Specifically, Van Veelen et al. [55] described that one of the two patients presenting with postoperative papilledema underwent biparietal re-expansion. Similarly, Mathijssen et al. [33] identified raised ICP as the primary indication for reoperation in 27 patients, although the diagnostic criteria were not uniformly based on invasive measurements but on fundoscopy, OCT, and clinical assessments. These findings highlight the potential for misclassification when relying solely on fundoscopic evaluation, which may lead to an overestimation of postoperative intracranial hypertension: papilledema, where reported in isolation or with clinical signs of intracranial hypertension, without direct ICP measurement, should be considered indicative of possible rather than definitive intracranial hypertension.

Strip craniectomy is proven to be an effective procedure with even superior cosmetic results to OCVR, and its minimally invasive variants have gained popularity due to their association with shorter operative times, lower blood loss, reduced hospitalization, and acceptable morphologic outcomes [17–19, 25, 30]. Endoscopic-assisted craniosynostosis surgery, introduced in the 1990 s by Jimenez and Barone, remains a relatively recent advancement with promising results [21, 22] with emerging evidence suggesting that it may still be effective in selected cases even without helmeting and in patients older than 6 months of age, expanding its potential indications and flexibility in clinical practice [2].

In this systematic review, endoscopic-assisted strip craniectomies (EASC), typically followed by helmet therapy, were generally associated with a very low incidence of postoperative ICP raise. In 4 studies, the authors did not report any case of raised ICP (Table 3) [14, 25, 27, 56]. In contrast, in the longitudinal study by Riordan et al., 12 patients (2.4%) developed secondary sagittal suture refusion, presenting clinical or radiological evidence of raised ICP, all of whom subsequently required a second surgery [41]. Aza et al. assessed multiple surgical strategies and reported that endoscopic-assisted procedures demonstrated the lowest complication and reoperation rates, while total vault remodeling and fronto-facial distraction were associated with higher morbidity [17]. Favorable outcomes could be related to the early age at surgery and consequent greater compensatory cranial growth [19, 38]. Moreover, the reduced surgical invasiveness and limited disruption of calvarium integrity likely preserve normal cerebrospinal fluid dynamics [18, 28].

Studies evaluating modified strip craniectomy or spring-assisted cranioplasty report a relatively higher incidence of ICP elevation (Table 3) [1, 31, 42, 51–53]. Van Veelen et al. described a cohort in which papilledema was observed in approximately 2.4% of patients treated with extended strip techniques [53]. In the study by Sadon et al., two patients developed raised ICP with papilledema and underwent secondary cranial expansion, whereas one patient exhibited borderline intracranial pressure and was successfully treated with conservative management [44]. As it occurred in our series, indeed, some children with papilledema at fundoscopy may harbor drusen or may remain asymptomatic without requiring a new surgery. Thomas et al. reported raised ICP after modified strip craniectomy in 14.6% of patients, while only 1.6% of the OCVR cohort experienced postoperative intracranial hypertension [51]. The lower rate of postoperative raised ICP shown in our series (1.5%) could result from the more extensive (or the “less mini-invasive”) surgical technique. Despite these concerns regarding long-term ICP control, especially in older age groups or when helmeting is not implemented rigorously, modified strip craniectomy and its variants continue to offer notable perioperative advantages [43]. Several studies, including those by Makoshi et al. and Math et al., have documented significantly lower intraoperative blood loss, shorter operative times, and reduced hospital stays compared to OCVR [5, 28]. Furthermore, when performed early in life and in combination with appropriate helmet therapy, strip craniectomy can achieve satisfactory anthropometric and cosmetic outcomes [29, 46, 47]. Although the risk of postoperative ICP elevation appears slightly higher in this group, late or incomplete correction, suboptimal helmet compliance or lack of standardized postoperative surveillance may also have influenced the results on the ICP increase.

These findings suggest that while endoscopic approaches may offer the most favorable profile regarding long-term ICP control, strip craniectomy remains a valuable and effective alternative in selected patients. Its continued use may be justified in clinical settings where endoscopic expertise or resources are limited, provided that rigorous follow-up and timely recognition of potential ICP-related complications are ensured.

Although OCVR is traditionally considered a definitive treatment in craniosynostosis, delayed raised ICP has also been reported following this approach. Moore et al. observed a 7.1% rate of late-onset raised ICP requiring second surgery, with higher incidence in patients operated before 6 months of age [33]. Similarly, Cetas et al. found a 6.2% rate of delayed ICP elevation in a cohort of 81 patients followed for at least 3 years [7]. In contrast, McClugage et al. reported a considerably lower incidence: only one patient out of 105 (0.95%) who underwent OCVR for isolated sagittal synostosis developed confirmed delayed intracranial hypertension requiring reoperation, but still emphasized the importance of long-term follow-up [32]. Therefore, delayed ICP elevation following OCVR is uncommon but not negligible, as demonstrated also by our personal experience, particularly in patients treated early in life and in those with sagittal synostosis (which may develop multisutural synostosis).

According to these figures, it can be deduced that, in spite of the lower rates of late postoperative intracranial hypertension in OCVR, mini-invasive procedures do not increase significantly the risk compared with more extensive ones. Several reasons can be advocated to explain this trend: (1) a mean earlier age at surgery in MIC and EASC vs OCVR, with the possibility to relieve in advance the venous compression exerted by the synostosis; (2) use of a postoperative helmet, with quicker and more effective reshaping of the skull with prevention of compression of the venous sinuses. This would justify the higher risk of postoperative raised ICP shown by strip craniectomies alone or spring-assisted reshaping; and (3) poor overall risk of recurrence of the monosutural craniosynostosis in general.

On the other hand, it should be acknowledged that the risk of postoperative raised ICP after mini-invasive surgery is not trivial. Once again, some hypotheses can be done: (1) incomplete control of ICP because of intrinsic limits of some techniques or suboptimal performance of the operation in some patients; (2) progression of the stenosis into a multisutural one in some instances, with or without evidence of genetic mutations; (3) the quality of the follow-up which seems to be longer and more accurate than in the past, leading to an apparent increase in the cases with postoperative raised ICP.

Unfortunately, the analysis of the risk of reoperation concerning the different types of craniosynostosis (Table 4) does not allow drawing conclusions on the role played by postoperative raised ICP on this risk. Actually, many studies do not specify the cause of the reoperation so that the reoperation risk reflects that related to the intrinsic characteristics of the synostoses (higher in uni/bicoronal or multisutural craniosynostosis). Only Thomas et al. reported systematic surgical revision in all cases with confirmed ICP elevation [51], whereas most series adopted a selective approach in these patients, reserving reoperation for persistent or symptomatic intracranial hypertension.

## Limitations

Across the included studies, methods for ICP assessment were notably heterogeneous, sometimes relying on indirect clinical signs, while others used imaging or fundoscopy, and only a few studies employed invasive monitoring. Furthermore, thresholds for intervention and duration of monitoring varied, underscoring the need for standardized diagnostic criteria. Moreover, 12 included studies were retrospective, which inherently increases the risk of selection, reporting, and observer bias. Finally, a possible, “intrinsic” limitation of this study comes from the short postoperative follow-up and the lack of systematic screening for raised ICP in some studies. Another major concern relates to the reliance on indirect markers of intracranial hypertension, such as papilledema, which may not reliably reflect true ICP values, particularly in younger patients, as fundoscopic examination, although widely used, has important limitations. Given this variability, cases of postoperative intracranial hypertension identified through indirect methods should be interpreted with caution, as they may reflect suspected rather than confirmed intracranial hypertension. This lack of uniform diagnostic criteria limits the comparability of results and may have influenced the reported incidence of postoperative ICP raise. 

## Conclusions

According to this systematic review, minimally invasive techniques are confirmed to be safe and potentially protective approaches against postoperative intracranial hypertension, although a certain risk of showing a postoperative raise of ICP is found. Such a risk is not significantly higher than in open calvarial remodeling. However, the lack of standardized ICP measurement methods and the variability in patient selection limit the strength of any definitive recommendations.

## Data Availability

No datasets were generated or analysed during the current study.
